# Exploring anal self-examination as a means of screening for anal cancer in HIV positive men who have sex with men: a qualitative study

**DOI:** 10.1186/1471-2458-14-1257

**Published:** 2014-12-11

**Authors:** Jason J Ong, Meredith Temple-Smith, Marcus Chen, Sandra Walker, Andrew Grulich, Christopher K Fairley

**Affiliations:** Melbourne School of Population and Global Health, University of Melbourne, Melbourne, Victoria 3010 Australia; General Practice and Primary Health Care Academic Centre, University of Melbourne, Carlton, Victoria 3053 Australia; Melbourne Sexual Health Centre, Carlton, Victoria 3053 Australia; Central Clinical School, Monash University, Clayton, Victoria 3168 Australia; Kirby Institute, University of New South Wales, Darlinghurst, NSW 2010 Australia

**Keywords:** Anal cancer, Digital ano-rectal examination, Self-examination, HIV, Men who have sex with men

## Abstract

**Background:**

Anal cancer is relatively common in HIV-positive men who have sex with men(MSM). However there are no clear guidelines on how to effectively screen for anal cancer. As earlier diagnosis of anal cancer is associated with increased survival, innovative ways such as utilizing anal self-examination to identify anal cancer should be explored.

**Method:**

Semi-structured interviews were conducted with 20 HIV-positive MSM from a range of ages (35 to 78 years). This study explored acceptability and barriers to implementing ASE as a method of anal cancer screening. Framework analysis was used to identify themes.

**Results:**

Seventeen out of 20 men had conducted an ASE before - six (35%) were for medical reasons, six (35%) for sexual reasons, three (18%) for both medical and sexual reasons, and two (12%) for cleaning purposes. Only 5 men were currently confident in detecting an abnormality. Whilst men were generally comfortable with the idea of utilizing ASE as a means for detecting anal cancer, potential barriers identified operated at three levels: attitudinal (discomfort with any anal examinations, anxiety about finding an abnormality, preference for health professional examination), knowledge (lack of awareness of anal cancer risk and ignorance of anal cancer symptoms) and practical (inadequate physical flexibility, importance of hygiene).

**Conclusion:**

ASE may be an acceptable means for anal cancer detection in HIV-positive MSM but training in detecting abnormalities is needed. The preference for health professional examination and inadequate physical flexibility may preclude its use for some men. Future trials to confirm its wider acceptability will be needed before undertaking an effectiveness trial for detecting anal cancer.

## Background

Anal cancer occurs at higher rates in men who have sex with men(MSM) compared with the general population [1] and at an even higher rate in HIV-positive MSM [2]. Incidence rates of up to 144 per 100,000 have been reported in several countries [3, 4], making it one of the most common cancers in HIV-positive MSM [5].

Anal cancer is associated with substantial mortality but early diagnosis is associated with considerably improved survival. In a case series of 38 HIV-positive men, those with tumours <3 cm had a 5 year cancer specific survival of 85% compared to zero in those with tumours >3 cm [6]. In a French series of 69 anal cancers diagnosed at <1 cm, the 5-year disease specific survival was 100% [7]. Data from USA showed that those with a localized cancer at diagnosis had a 5-year survival rate of 82% compared to 36% to 59% when the cancer was not localized [8]. These data highlight the importance of early diagnosis.

Currently anal cancers are usually diagnosed late with an average size of 2.9 cm at diagnosis in a HIV-positive population [9]. In one study symptoms were present for an average of 22 weeks before men presented to a health care provider. Many experts suggest that implementing early detection of anal cancer using regular digital ano-rectal examination(DARE) by doctors should be routine in those at highest risk (i.e. HIV-positive MSM) [10–12]. The term DARE has been used specifically for anal cancer studies to emphasize the importance of feeling the anal canal as part of the examination [13]. This also includes an examination of the perianal region (the area of skin extending up to 5 cm from the anal verge). A recent study of 138 HIV-positive MSM with anal cancer found that early anal cancer detection was possible if men were closely followed up with regular DARE by a health professional [13]. However it is not known if anal cancers may be detected earlier and more frequently by encouraging self-examination in addition to regular DARE by a health professional. Self examination has been investigated for breast cancer [14], skin cancer [15], and testicular cancer [16]. Although self-examination provides a means for early detection, the recommendation by health authorities for self-examination remains controversial and inconsistent. For example, although many women find their own breast cancers, in countries with established breast screening programs, breast self-examination is unlikely to provide additional benefits in decreasing breast cancer mortality [17]. It is important therefore to determine the settings where self-examination may still provide clinical benefit. Given that there are no established anal cancer screening programs in many countries and that anal cancers are currently diagnosed late [9], teaching men to be aware of symptoms and changes in their anus may lead to earlier presentations of anal cancer. An anal self-examination(ASE) would involve teaching men to feel the skin in the peri-anal area and to perform a self-DARE, palpating for a lumps or ulcer. If ASE was to work effectively it would be necessary as part of an education program that patients were strongly encouraged to consult a doctor if any new lesions of concern were identified.

Before ASE can be tested in the population for its effectiveness in detecting anal cancer, the views on the acceptability and feasibility of ASE from those at highest risk for anal cancer (i.e. HIV-positive MSM) are essential. This study is the first to determine men’s views of ASE as a means for detecting anal cancer.

## Methods

### Participants

Semi-structured face-to-face interviews were conducted with HIV-positive MSM recruited from a sexual health centre and a high HIV case-load general practice in Melbourne, Australia. To ensure maximum variation sampling, a quota of up to 5 men from each of four age categories were selected: 35–44, 45–54, 55–64 and 65 years of age or older. Men with past or current anal cancer, or who were not fluent in English were excluded.

### Procedure

During a consultation recruiting doctors gave eligible men an information and consent form, and invited participation in the study. The researcher(JO) then contacted the patient to answer further questions and to organize a convenient time for the interview. Written consent was obtained at the time of the interview. The interviews were recorded for later transcription and thematic coding.

Structured questions captured participants’ demographic data and the remainder of the interview was semi-structured with open questions aimed at eliciting views on ASE, the barriers and facilitators for implementing ASE as a means of anal cancer screening. An ASE was defined as self-examination of both the anal canal and perianal region. A manikin (Rectal examination Trainer Mk 2, part no: 60120, http://www.limbsandthings.com) and pictures (http://www.anal.org.au/clinician/resources/Gallery/21acancer_acancer.html) were shown to participants so to help visualize where the perianal region was and what potential cancers may look like. As this was an exploratory study on the attitudes towards ASE, we did not conduct any education on how to feel for an anal cancer. Interviews were undertaken until no new insights were captured i.e. data saturation was achieved.

### Analyses

Data were transcribed, organized electronically and assigned codes using NVIVO (QSR International Pty Ltd, version 10.0, 2012), a qualitative research software program. Data were analyzed using an iterative approach. After each interview a preliminary analysis was performed to allow identification of new themes emerging from the interviews to be included in subsequent interviews. Once all interviews were completed, content analysis was performed to block, group and label the data to identify emerging themes. We looked for differences in themes between older and younger participants, and those who had performed ASE to look for anal abnormalities compared to those who performed ASE for sexual reasons only. Coding was conducted independently by two researchers (one sexual health clinician and one sexual health researcher) and then discussed with the research team to achieve consensus on common themes. Finally, coding for themes and concepts was used to frame the remaining data. We adhered to the qualitative research review guidelines (RATS) [18] in reporting the findings of this research. This research was approved by the Alfred Health Human Ethics Committee (Project 32/13).

## Results

Of 22 men approached, two declined (one was too busy, and the other not interested). Participants’ demographics are summarized in Table 1. Men described themselves as generally healthy(mean score of 75) on a general scale of 0 to 100, where 100 equated to perfect health. They were a relatively pro-active group with many describing exercise and dietary changes as part of their health maintenance. Seven men practiced testicular self-examination and three men skin self-examination on an ad hoc basis.

**Table 1:** **Demographics of participants**

	Number of participants (total = 20)
Born in Australia	18 (90%)
Healthcare card holder*	12 (60%)
Private health insurance	4 (20%)
Education	
- Primary Education	1 (5%)
- Secondary Education	4 (20%)
- Technical and Further Education	5 (25%)
- University degree	7 (35%)
- Postgraduate	3 (15%)
Employment	
- Work full time	6 (30%)
- Work part time	3 (15%)
- Unemployed	3 (15%)
- Retired	8 (40%)
Currently on antiretrovirals for HIV	19 (95%)
Past anal issues	
- Sexually transmitted infection	2 (10%)
- Haemorrhoids	4 (20%)
- Warts	5 (25%)
- Fissure	1 (5%)
- Perianal abscess	2 (10%)
Mean age = 54.5 years (range 35–78)	
Mean years living with HIV = 18.8 years (range 4–32)	

*Healthcare cards are given to Australian residents with a lower income to be able to access cheaper medicines and medical costs.

### The practice of ASE

Seventeen out of 20 (85%) men had inserted their finger into their anus before – six (35%) were for medical reasons, six (35%) for sexual reasons, three (18%) for both medical and sexual reasons, and two (12%) for cleaning purposes. Currently none were doing this on a regular basis to screen for anal cancer. Only a minority (5 men) felt confident in detecting an abnormal lesion. Four men had found abnormalities themselves and presented to the doctor for further examination (1 skin tag, 1 scarring, 2 haemorrhoids).

Men described how they would conduct an ASE. The location of performing an ASE would either be in the shower, bathroom or bedroom. The positions adopted did not appear to be related to the age of participants and varied (from most frequently used to least): (lying on back or side, squatting, standing, sitting on the toilet, kneeling). The majority of men would use some sort of lubricant in the process (13 men would use KY jelly, 2 use soap) and a minority (4 men) would also use a glove. Four men would use a mirror to also try to visualize the peri-anal area.

### Discussion of anal issues

The majority of men felt comfortable raising anal issues within a medical setting. Some men expressed that the ease of raising the issue stemmed from their sexual identity.

In a medical context I have absolutely no problem at all, none whatsover… (62 years old, living with HIV for 7 years)

It’s part of the gay men’s life, you know. (47 years old, living with HIV for 11 years)

A minority of participants stated that comfort levels may alter depending on whether the doctor was their usual HIV doctor and the gender and/or sexual orientation of the doctor.

I’d been seeing my same doctor now for probably 15 years, so, no I don’t feel uncomfortable talking to him. If it was a general GP probably a different story. (49 years old, living with HIV for 29 years)

I think that an HIV doctor, and also a gay doctor, has a much better understanding of what people risk being gay. (55 years old, living with HIV for 32 years)

I always have found those personal things easier to talk about with a female doctor. (47 years old, living with HIV for 28 years)

Some participants recognised that underlying their choice of doctor with whom to discuss anal issues was a trusting relationship.

With another doctor - as soon as I’ve got some sort of relationship - then I think, for me, those things are easier to talk about. (47 years old, living with HIV for 28 years)

Despite the majority of men being comfortable in addressing anal issues with a health professional, only two men reported that their doctor had raised the issue of anal cancer. As a result of the paucity of discussions around anal cancer, the understanding of anal cancer was poor amongst participants.

I don’t know much about it at all other than it is a type of cancer and it’s probably not a good way to die. (56 years old, living with HIV for 15 years)

Participants reported that the majority of doctors would only discuss anal issues if the participant specifically brought the topic up.

It’s more about problem solving [for doctors] (55 years old, living with HIV for 32 years)

Indeed the majority of participants suggested that doctors should be more pro-active in addressing anal health.

Most people are going to find it awkward, so until someone raises it with you, you’re probably never going to do it yourself unless there’s a huge problem, and by then it might be a little late. (39 years old, living with HIV for 13 years)

Many men suggested the manikin was unnecessary for showing them what is meant by the perianal region, but a stronger reaction to the pictures of anal cancer was obvious.

That’s nasty! (47 years old, living with HIV for 21 years)

It should make anyone, you know gay or not, examine themselves (47 years old, living with HIV for 11 years)

### Potential barriers for implementing ASE

Despite enthusiasm for considering ASE, there were barriers expressed under the themes of attitudes, knowledge and practice (Table 2).

**Table 2:** **Themes of barriers to anal self-examination (ASE)**

Attitudinal barriers	Concern about discomfort with any anal examinations
	Anxiety about finding an abnormality
	Preference for a health professional to do an anal examination
	Too many sensations or sexual connotations with ASE
Knowledge barriers	Lack of awareness of anal cancer risk
	Ignorance of how anal cancer presents
Practical barriers	Inadequate physical flexibility to conduct an ASE
	Importance of cleanliness before ASE

#### Attitudinal barriers

Enthusiasm for ASE was especially evident in younger participants, who saw ASE as simple and who approved of the concept of early detection.

Women do breast checks for themselves and discover their own lumps… there’s no reason why a man can’t do the same thing. (47 years old, living with HIV for 21 years)

Any type of early detection is great, from reports you hear about people like breast examinations… They catch it early and it’s a lot better for them. (47 years old, living with HIV for 8 years)

Everybody should be doing it, no matter what part of the body it is. I mean everyone should be keeping an eye out for any changes… self-examination for me it’s a must. It’s a must. (42 years old, living with HIV for 4 years)

Despite perception of some discomfort, most men conveyed that they would still allow an anal examination if there were clear benefits.

There is something about a digital examination that feels more intimate… and I vaguely feel a little bit uncomfortable with a doctor doing that. But not enough to stop me doing it. (43 years old, living with HIV for 7 years)

However there was agreement that not all men might be comfortable with any sort of anal examinations.

Some are very, you know, “Don’t come near my [anus]” …That’s just something that they’ve got a phobia against or they’ve been brought up thinking no, no one touches that spot, you know… If you go anywhere near them down there and they clench like - - you know, clamp. (47 years old, living with HIV for 11 years)

For men who have never performed an ASE to look for anal abnormality before, they discussed the potential for anxiety if an abnormality was detected. Interestingly, this was always spoken of in the third person, as if to distance themselves from such anxious men.

The only downside is making people slightly paranoid potentially. But ultimately I think most people - particularly people who are in a high risk category it’s probably better to err slightly on the side of paranoia [laughs]. (40 years old, living with HIV for 4 years)

Maybe some people get a haemorrhoid and they get hysterical and think they’re going to die or something because they don’t know what it is. (61 years old, living with HIV for 8 years)

This anxiety seemed to stem predominantly from recognition that following an abnormal ASE further investigation would be required.

The waiting period between the doctor that refers you to somebody that is more the stress period for me, because I’m going “Well the doctor thinks they need to refer it”, then you know that there’s potentially something. (40 years old, living with HIV for 4 years)

The men who had not performed ASE to look for abnormality before preferred anal examinations to be performed only by a medical expert, not themselves. Underlying this ‘hands-off’ attitude was a fear of missing something or the preference for a health professional to decide whether something is normal or abnormal. In addition some men stated that conducting an examination with their own finger would be too difficult as it may lead to too many sensations and had sexual connotations.

I don’t think it’s up to me to decide whether something is normal or not, it’s up to the doctor. (56 years old, living with HIV for 15 years)

I couldn’t do it myself. What if I miss something… [ASE is] not your normal medical procedure. It’s more of a sexual thing for a gay person. It would turn on a whole lot of ranges of feelings and sensations as well. Sexual desires, all that kind of stuff. (49 years old, living with HIV for 29 years)

#### Knowledge barriers

Two knowledge barriers were noted – lack of awareness of anal cancer risk and ignorance of what to look for during ASE. There was frustration amongst participants that they did not have enough information.

It’s not out there. I can tell you it never crossed my mind that I should be looking for anal cancer…. If you have that knowledge then you can act on that knowledge but if you don’t know, well you just don’t know. (62 years old, living with HIV for 7 years)

When you get to a certain age there’s quite a lot of talk about prostate cancer, not a lot about anal cancer. (61 years old, living with HIV for 8 years)

A result of this lack of awareness may be a tendency to ignore the link between anal symptoms and potential anal cancer.

They would probably brush it off thinking, “Oh, no, it will never happen to me” but in reality that could be quite the opposite. (47 years old, living with HIV for 21 years)

I know of cases where people ignored it… and had been told… “If you’d come three months ago, your chances would have been much, much stronger.” (72 years old, living with HIV for 26 years)

Furthermore, the lack of knowledge led to misconceptions of risk factors, misperception of personal risks and assumptions of an association between anal cancer and other cancers.

A lot of men who would categorise themselves as being active [insertive anal sex] rather than passive [receptive anal sex], they would pigeonhole themselves and say “Well, that doesn’t affect me”. (39 years old, living with HIV for 13 years)

[My risk of anal cancer would] be low because I don’t really have a sexual partner any longer. (78 years old, living with HIV for 31 years)

[Anal cancer] seems to be associated with having partners of different strains of semen from what I’ve read… I’m a great believer in hygiene before and after sex by douching. [Facilitator: You think that helps?] Yes, by cleaning oneself. (69 years old, living with HIV for 28 years)

My grandfather died of bowel cancer… my father died of prostate cancer… but because they’re sort of in the same area, it sort of makes me think maybe I’m more likely to be a candidate for anal cancer. (47 years old, living with HIV for 28 years)

Although men felt comfortable in noticing a change in their anal region, there was no specific understanding of what they should be looking for when they examined themselves.

I never know quite what I’m looking for but I would recognise a change if there was inflammation or change of colour or anything like that. (72 years old, living with HIV for 26 years)

I don’t quite know what I’m looking for – I don’t quite know what the sensation of that difference is. (61 years old, living with HIV for 8 years)

#### Practical barriers

Whilst largely supportive of ASE, men acknowledged that there may be a few practical barriers to an adequate examination, even for younger participants.

It’s in an obscure spot. It’s not visible… it’s in an enclosed area. So it can be hard to look after if something goes wrong. (47 years old, living with HIV for 11 years)

Whilst a few expressed potential issues with the physical flexibility needed for an adequate examination, the majority (including older participants) believed that flexibility was not a major barrier.

When I was younger, I was quite flexible and reaching around and examining myself was quite - it was easy to do, because I was always constantly checking for warts… the external part of it would be quite easy to detect. But the internal part I find would be a little bit more difficult. (49 years old, living with HIV for 29 years)

Everything’s harder when you get older, really… but I don’t find [ASE] a lot harder. (72 years old, living with HIV for 26 years)

A minority of participants expressed a concern around hygiene prior to ASE. For these men, it was important for them to be completely clean before they would conduct an ASE.

I don’t go anywhere near somebody else’s or my own butt… without it being clean… I mean, not only is it unhealthy, it’s unpleasant and undignified. (55 years old, living with HIV for 32 years)

This is where I probably would get uncomfortable if I knew I hadn’t been to the toilet or wasn’t clean or something. (40 years old, living with HIV for 4 years)

There’s that obsessiveness about being unclean, I think maybe more so being a gay man. (47 years old, living with HIV for 28 years)

## Discussion

Whilst self examination has been investigated for screening of other cancers [19–21], this is the first study to explore ASE as a means for anal cancer detection. If ASE can detect anal cancers earlier than its current average size of 2.9 cm at diagnosis [9], there may be a possibility to avoid chemoradiation and its associated comorbidities. The latest National Comprehensive Cancer Network Clinical Practice Guidelines in Oncology suggest that local excision without chemoradiation should be offered for well differentiated anal cancers <2 cm in the perianal area [22]. Therefore, exploring men’s current practices and the feasibility and acceptability of ASE is an essential first step before evaluating its effectiveness in anal cancer detection. This is the first study to demonstrate that ASE may be feasible and acceptable to HIV-positive MSM.

The most enthusiastic group for ASE were the younger participants who wanted to be more proactive with their health. The least enthusiastic group for ASE were those who had not performed ASE to look for anal abnormality before. This group were those who raised the issues of anxiety about finding an abnormality, preference for a health professional to conduct the anal exam, and believing that too many sensations or sexual connotations would distract them from an effective ASE. This highlights that ASE may not be for everyone. It is particularly important to identify men who may potentially experience adverse psychological impacts through screening [23] as the difficulties in performing ASE may outweigh the benefits in some men. In this case an alternate cancer screening program may be more realistic or acceptable for the patient - for example an annual DARE performed by the clinician. Indeed our study emphasizes that there is not ‘one size that fits all’.

Despite the majority of men describing comfort in discussing anal issues with a health professional, very few men reported any health professional initiating discussion about anal cancer with them. There must be more discussion about anal cancer and how it can be screened as there continues to be poor understanding of anal cancer in the MSM community [24]. In fact, participants of the study expressed a desire for their doctor to proactively discuss their risk for anal cancer and how to recognize symptoms that warrant an examination by the doctor. A recent review of increasing colorectal screening uptake emphasized the link between screening participation with the participants’ knowledge and risk perception of the cancer [25].

To ensure an adequate examination of the whole anal canal, doctors should discuss the practical issues of physical flexibility and hygiene. Certain men may not have adequate flexibility to conduct an ASE and an alternative may be agreed upon e.g. teaching patients to have a low threshold for an anal examination by the doctor if they noticed any abnormal symptoms (like bleeding, pain or discharge) [9]. For men performing ASE, it may be important at this stage to encourage all men to present to a HIV doctor if any new lesion is detected because it is still not clear what are the specific characteristics of an early anal cancer. Anal cancers may be described as a palpable mass, induration or ulcer [9, 13]. If the doctor is uncertain of the diagnosis, a biopsy of the lesion is possible to distinguish an early cancer from other anal pathologies.

The strength of the study was the use of qualitative methodology which allowed in-depth exploration of men’s views about this sensitive issue, and which allowed identification of potential barriers of ASE. A limitation of the study was that participants were already highly engaged with a HIV medical service and largely described themselves as healthy, pro-active men. Future studies should aim to include men who are not regularly engaged in medical care.

A quantitative study is needed to evaluate the practice and acceptability of ASE in HIV-positive MSM. A larger study population may offer enough power to detect differences in the likelihood of using ASE (e.g. are those who have had previous anal conditions or received screening for anal dysplasia more comfortable to use ASE); and the likelihood of detecting anal cancer at an earlier stage compared with those not practicing ASE.

## Conclusion

In this exploratory study, ASE was seen as feasible and an acceptable means for anal cancer detection in the majority of participants. For those comfortable with conducting ASE, further evaluation of the best methods for teaching anal cancer detection to participants is needed. Potential barriers (including preference for health professional examination and inadequate physical flexibility) may preclude its use for some men because these barriers are not rectifiable. If ASE was shown to be widely acceptable, further studies will be needed to confirm its cost-effectiveness for reducing the morbidity and mortality of anal cancer through earlier presentation of anal cancer.
